# Microfilariae coexisting with a follicular lesion in thyroid aspirate smears in an uncommon case of a retrosternal thyroid mass, clinically presenting as malignancy

**DOI:** 10.4103/1742-6413.76732

**Published:** 2011-02-12

**Authors:** Bharat Rekhi, S. V. Kane

**Affiliations:** Department of Pathology, Department of Cytopathology, Tata Memorial Hospital, Parel, Mumbai, India

To the Editor,

Filarial infection is a public health problem in the tropical regions of South-east Asia, including the Indian subcontinent that includes certain areas of heavy infestation, like Madhya Pradesh, Bihar, Jharkhand, along with others. It is caused by the nematodes *Wuchereria bancrofti, Brugia malayi, Brugia tumori* and others, transmitted through a mosquito *Culex fastigens*. Its disease manifestations include lymphoedema and superficial swellings.[1–3] Varied sites of involvement documented on aspirate smears include breast, epididymis, spermatic cord, lungs, etc.[4–6] Over the years, it has been uncommonly identified in the thyroid.[7–13] Herein, we present an uncommon case of a retrosternal thyroid mass that clinically presented as malignancy and on aspiration cytology disclosed a follicular lesion with coexisting microfilariae.

A 55-year-old gentleman from Ranchi (Jharkhand, India) was referred to our hospital with complaints of anterior neck swelling since 2 months, associated with dysphagia to solids and alteration of voice for a month. He had been a tobacco chewer for 30 years. He also disclosed history of aspiration a month ago, along with breathlessness. On clinical examination, his general condition was good. On local examination, a soft swelling was noted over his right thyroid and isthmus and a 10×7 cm hard swelling was noted over his left thyroid, extending into the retro sternum that did not move with deglutition. Left vocal cord palsy was noted. There was no stridor. Clinically, he was presumed with a diagnosis of stage IV carcinoma thyroid.

He underwent laboratory and radiological investigations, including an outside computed tomography (CT) scan, followed by fine-needle aspiration cytology (FNAC) on three occasions, including twice at our hospital.

His hemoglobin was normal i.e. 12 gm/dl (Normal: 13-17 gm/dl). Total leukocyte count was high i.e., 13.7×10e9/L (Normal: 4-10×10e9/L), including absolute neutrophilia. Percentage and absolute eosinophil counts were normal 4.73% and 0.65×10e9/L (Normal: 1-6% and 0.2-1×10e9/L). Basophil percentage was marginally high i.e., 0.33% (Normal: 0-0.2%). His thyroid function tests were within normal limits. There was no access for thyroid scan results.

Limited accessible imaging findings included a CT scan report that disclosed a left thyroid lobe lesion, eroding the thyroid cartilage, trachea and thoracic inlet. His chest X-ray was normal.

FNAC was performed by multiple passes in the thyroid using a 23-gauge needle. Smears were fixed in alcohol stained with Papanicolaou (Pap), while air-dried smears were stained with Giemsa stain. The initial smears were reported as papillary carcinoma thyroid. Repeat smears were hypercellular, comprising benign appearing follicular cells in focal acinar formations, flat monolayered sheets and focal papillaroid arrangements, lacking anatomical edge. Cells exhibited focal nuclear overlapping, focal grooves and fine chromatin clearing in rare cells. No nuclear elongation, uniform nucleomegaly or intranuclear inclusions were noted within the hypercellular smears. Besides, there were sheets of cystic macrophages and Hurthle cells in a background of thick and thin colloid. Diagnosis of an atypical follicular lesion of undetermined significance was considered. On critical examination, microfilariae of *W. bancrofti* were identified that displayed presence of sheath and were deficient in somatic nuclei at the posterior end of the body that was curved. A few rhomboid-shaped Charcot Leyden (CL) crystals were noted. There were no inflammatory cells. The previous smears were reviewed and a final diagnosis of microfilarial infestation, along with follicular lesion (of undetermined significance) was offered. [Figure 1a–e]

**Figure 1:**
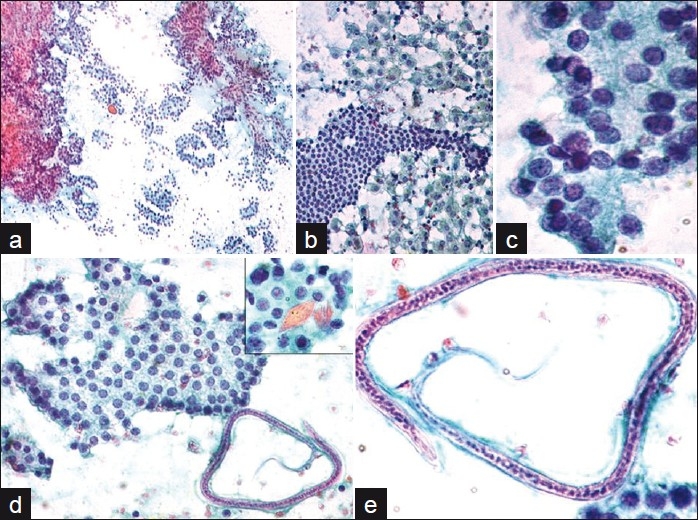
a) Hypercellular smear showing clusters of follicular cells. Pap stain ×100; b) Follicular cells with surrounding macrophages. Pap stain ×400; c) Cells showing focal nuclear overlapping. Pap stain ×1000; d) Flat monolayered sheet of follicular cells with adjacent microfilaria. Pap stain ×400. Inset showing Charcot-Leyden crystals. Pap stain ×1000. Pap stain ×1000; e) A single microfilaria of *Wuchereria bancrofti* with sheath and somatic nuclei. The anterior end (thin arrow) and curved posterior end (thick arrow), lacking somatic nuclei seen. Pap stain ×1000

Following, the patient succumbed to death within a week. However, post-mortem examination could not be performed.

## DISCUSSION

The present case is a rare manifestation of filarial infection that has been uncommonly documented in the thyroid. Most of the reported cases revealed presence of colloid or nodular goitre, except for an occasional case of malignancy and another case of a follicular neoplasm.[7–13] In view of hypercellularity, papillaroid formations, along with presence of focal nuclear overlapping and grooves, the initial diagnosis in the present case was that of a papillary carcinoma. However, on detailed evaluation on review, only some of these features were noted in rare cells. The intranuclear inclusions were lacking within the hypercellular aspirate, along with presence of macrophages and Hurthle cells against a background of colloid. Hence, as per The Bethesda System for reporting thyroid cytopathology guidelines, the present case fitted in the category of follicular lesion of undetermined significance.[14] In addition to a follicular lesion, the second aspirate revealed microfilariae of *W. bancrofti*. Besides, CL crystals, as reported in our earlier documented case of breast filariasis, were also noted.[5] However, there was no accompanying inflammation. The peripheral blood smears did not exhibit eosinophilia as noted by others[7,10,12,13] in their case descriptions. However, some investigators[8,9] similar to our case, did not identify eosinophilia in their case reports on microfilaria in thyroid.

The present case was amicrofilaremic as there was lack of parasite in the blood smear that also ruled out contamination from blood. The possible explanation for this unusual occurrence is the lodgment of the parasite in the thyroid vasculature, including lymphatics. This patient hailed from an area with high endemicity, both for goiter and filariasis.[2] A similar observation was noted by Sodhani *et al*,[7] wherein the described case came from the area with high endemicity for both, goitre and filariasis.

## CONCLUSION

FNAC is extremely useful in identifying filarial infection at uncommon sites like thyroid. An index of suspicion is necessary, especially in cases coming from high endemicity to tertiary cancer referral centre like ours’. A timely diagnosis that might be possible with FNAC could possibly help in obviating surgery in such cases that are treated with drugs, except in a case like the present one with a retrosternal mass, where surgery was required in order to relieve pressure symptoms. However, lymph node dissection would have been avoided, unlike in a case of thyroid carcinoma. The final histopathological examination could not be performed as the patient succumbed to death and autopsy was not performed. The possible reasons could have been related to pressure symptoms from the retrosternal mass, as filarial infection is rarely fatal. A coexisting hypercellular follicular lesion on thyroid aspirate smears in the present case created a diagnostic challenge.

## COMPETING INTEREST STATEMENT BY ALL AUTHORS

We declare that we have no competing interests.

## AUTHORSHIP STATEMENT BY ALL AUTHORS

Each author acknowledges that this final version was read and approved. All authors qualify for authorship as defined by ICMJE http://www.icmje.org/#author. Each author par-ticipated sufficiently in the work and takes public responsi-bility for appropriate portions of the content of this article.

## ETHICS STATEMENT BY ALL AUTHORS

This manuscript is a case description in form of a letter. Therefore, IRB approval was not mandatory.

## EDITORIAL / PEER-REVIEW STATEMENT

To ensure integrity and highest quality of CytoJournal publications, the review process of this manuscript was conducted under a double blind model(authors are blinded for reviewers and reviewers are blinded for authors)through automatic online system.
